# Awareness and Attitudes Towards Telemedicine Among Medical Students in the United States

**DOI:** 10.7759/cureus.11574

**Published:** 2020-11-19

**Authors:** Steve S Kong, Azin Azarfar, Amnie Ashour, Christopher Atkins, Neha Bhanusali

**Affiliations:** 1 Medicine, University of Central Florida College of Medicine, Orlando, USA; 2 Internal Medicine, University of Central Florida College of Medicine, Orlando, USA; 3 Cardiac/Thoracic/Vascular Surgery, Renaissance School of Medicine at Stony Brook University, State University of New York, Stony Brook, USA; 4 Pediatrics, University of North Carolina, Chapel Hill, USA; 5 Rheumatology, University of Central Florida College of Medicine, Orlando, USA

**Keywords:** telemedicine, telehealth, medical student, medical education

## Abstract

Introduction: Telemedicine (TM) or telehealth is defined as the delivery of healthcare services at a distance using electronic means. It is a rapidly growing field of medicine that uses telecommunication to provide healthcare services to patients such as the elderly and those in rural locations who may otherwise be unable to make it to the hospital or physician’s office. With the rise in the popularity of TM, educating future physicians on this technology will become vital. This study aimed to explore medical students’ experiences and opinions regarding TM.

Methods: An online survey was sent to 287 medical students in 20 different allopathic medical schools in the United States. The survey consisted of 14 questions that included demographic information, information regarding TM exposure, interest in TM, and plans for future utilization.

Results: The result of this study indicated that only 17.4% of medical students had prior exposure to TM. However, the increased exposure to TM helped not only to increase awareness of the technology but also helped students form opinions on TM. Lastly, students in all intended specialties had interests in utilizing TM in the future with specialties such as pathology, psychiatry, ophthalmology, and dermatology indicating the highest levels of interest.

Conclusion: As medicine continues to incorporate technology into the care of patients, training institutions need to expose future physicians to the modalities of care they will be utilizing. The results of this survey suggest that the development of education and exposure to TM will become increasingly important as more medical students indicate interest in utilizing this technology.

## Introduction

Telemedicine (TM) serves as a digital platform for medical professionals to provide healthcare services to the patients remotely. It has roots that go far back hundreds of years, with the first documented telehealth encounter taking place in the late 1800s [1]. TM is defined as the transmission of medical information from one site to another via electronic communications to improve a patient’s clinical health status [2]. Despite the many barriers, there are multiple factors driving the growth and diffusion of TM: value-based healthcare; development of new products and technologies such as speech recognition, automation, artificial intelligence, and 5G wireless technology; TM-friendly regulation, policy, and reimbursement; and increasing TM adoption by employers, hospitals, and payers/health insurance.

Studies published in 2017 depicted that about 33% of respondents among US healthcare providers stated that half or more of their patients continued using TM services after their initial visit [3]. Additionally, there has been an increase in the number of patients using TM services in the United States, from less than 350,000 in 2013 to seven million in 2018 [4].

The rapid growth of TM has exposed the lack of training available in the United States to prepare clinicians for this modality [5]. Medical students have been shown to graduate feeling unprepared to utilize TM effectively [6]. The literature suggests that universities need to educate and teach medical students to use technology to improve the quality of patient care [7,8]. Nowadays, with new technologies and innovations, medical students are more likely to use computers, phones, and mobile applications [8]. However, this does not imply that they are comfortable using telehealth [7]. Zayapragassarazan and Kumar conducted a study that revealed that the awareness, knowledge, attitude, and skills among health professionals in regard to TM were not adequate and it highlighted the need to educate and train the practicing physicians, residents, medical students, and other health professionals about TM and issues related to its use, such as privacy concerns and ease of use [9]. On the other hand, patients are also willing to utilize modern technology as it is easier and more accessible to them [6]. In the United States, it has been shown that TM is becoming a societal and cultural trend [6]. Based on multiple studies from the past few years, most patients reported high satisfaction with their telehealth experience [8,10-12].

Furthermore, TM has proven to be a vital tool in the education and training of healthcare professionals. Various studies have demonstrated how telehealth is as effective as or more effective than traditional learning methods [13,14].

With the continued growth and utilization of TM, more studies need to be done assessing the effectiveness of TM curricula across medical schools in the United States. Examining medical students’ interest in TM could provide valuable insights regarding possible opportunities to supplement the curriculum of medical schools. This study aims to explore medical students’ experiences with education regarding telemedicine. More specifically, it sets out to answer these three questions:

“How interested are US medical students in telemedicine?”

“Do US medical students plan to utilize telemedicine after completion of medical school?”

“How does US medical students’ intended residency impact their interest level in telemedicine?”

## Materials and methods

An online survey study was conducted for first- through fourth-year medical students from 20 allopathic US medical schools. The survey was distributed via a Qualtrics link (Qualtrics, UT, USA) via email to different faculty representatives at different medical schools who then sent the surveys to the medical students. The study was approved by the Institutional Review Board at the University of Central Florida with the file number of SBE-16-12074. Informed consent was obtained by all participants prior to the start of the online survey.

The survey was researcher-designed for the purpose of the present study and it was sent to nearly 3500 medical students in all classes who were eligible to participate. We received a response rate of 8.2%. Inclusion criteria were all adults over the age of 18 years and enrolled in a US medical school. Those who did not complete the survey were excluded from the study.

Questions were modified from a previously used survey by Glover et al. [15]. The survey platform as a prospective study was conducted through Qualtrics, an online survey instrument, used for distribution and data collection. To determine statistical significance and justify any cross-tabulations, we utilized the Pearson chi-square test, likelihood ratios, and linear-by-linear associations.

**Figure 1:**
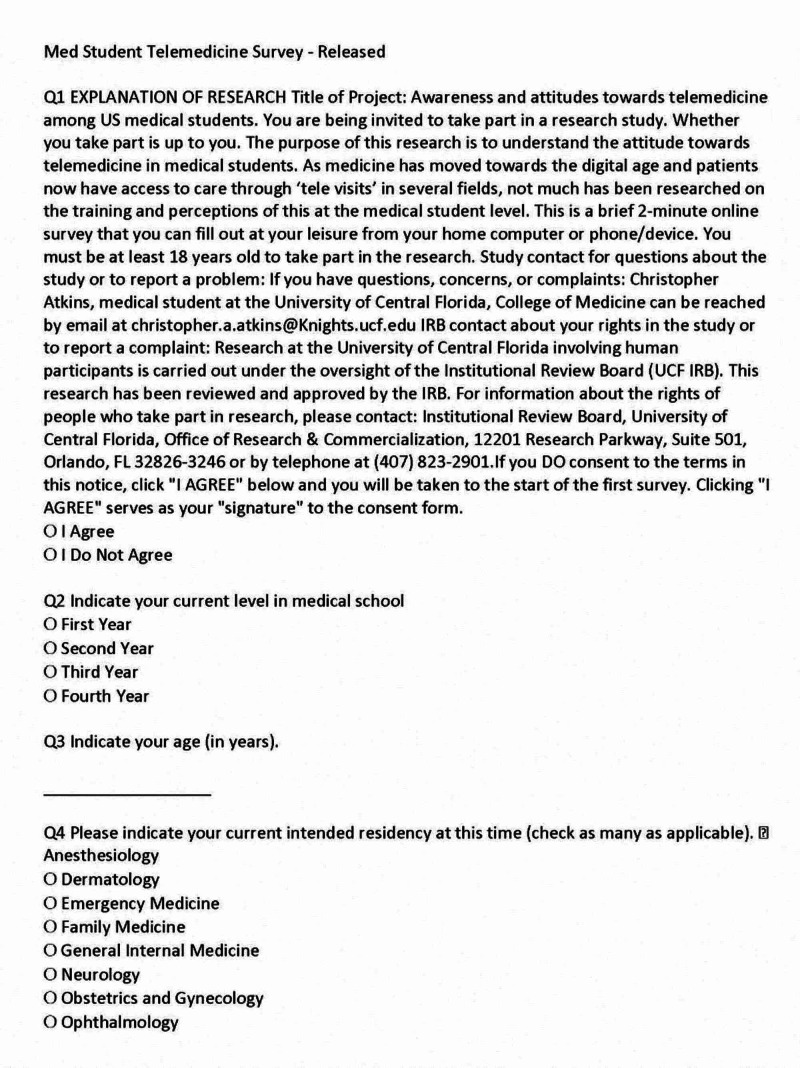
Medical Student Survey, Page 1

**Figure 2:**
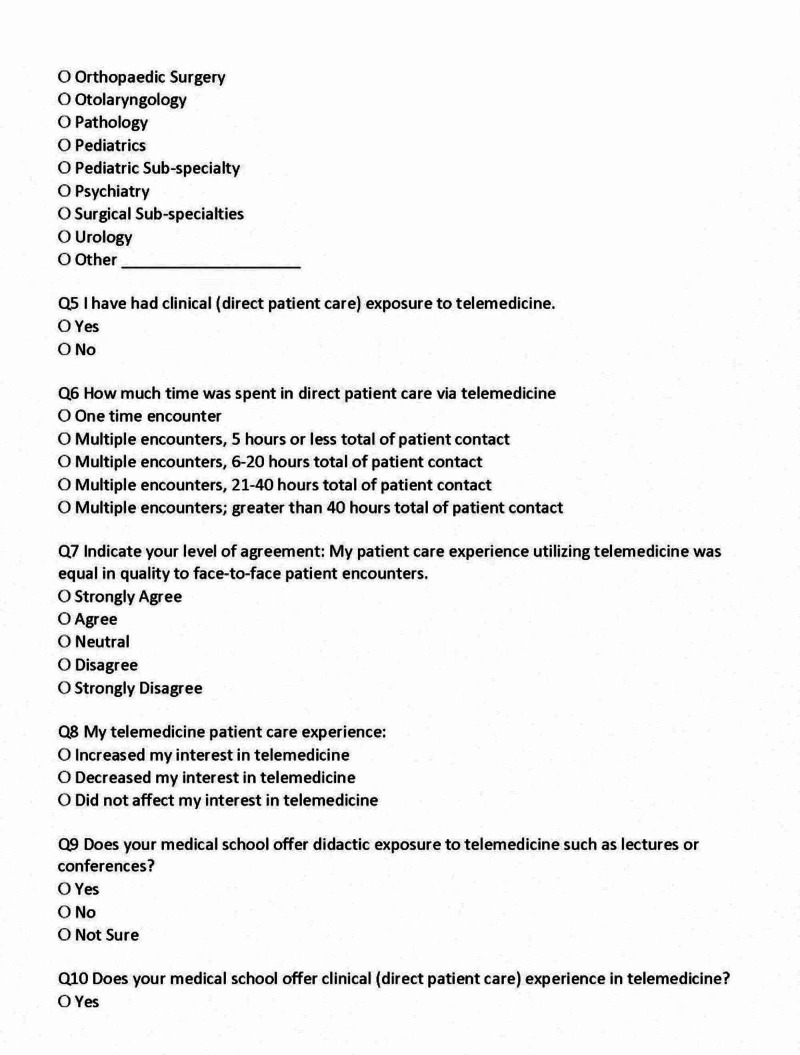
Medical Student Survey, Page 2

**Figure 3:**
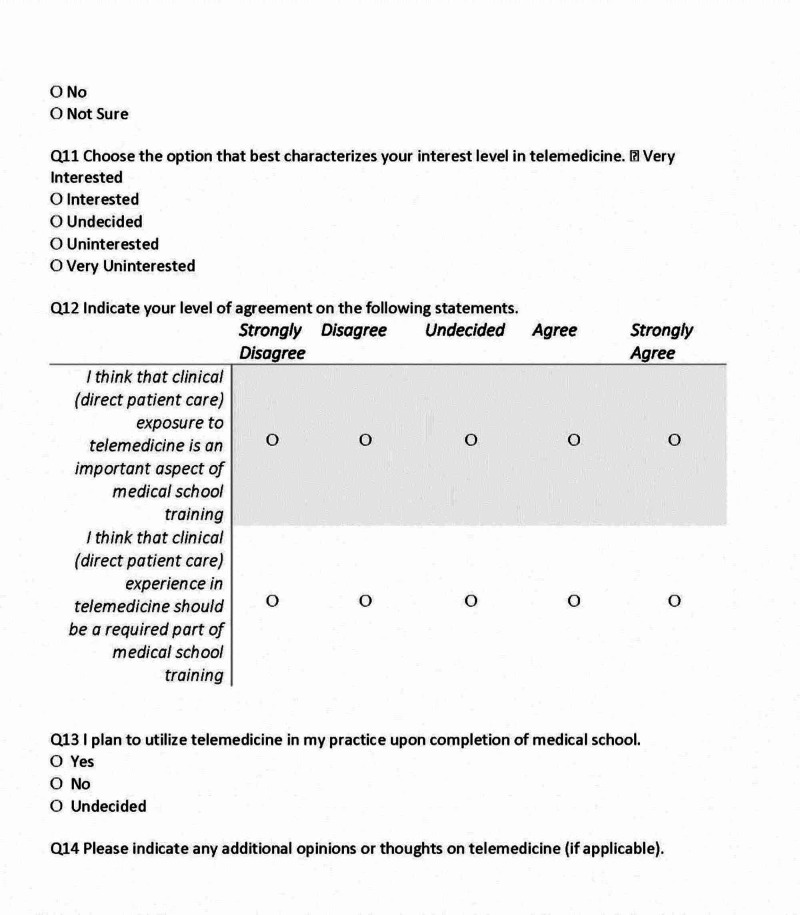
Medical Student Survey, Page 3

The survey consisted of 14 questions that included demographic information (four items), information regarding TM exposure (six items), interest in TM (two items), and plans for future utilization (two items) (Figures 1, 2, 3).

Statistical Package for the Social Sciences (SPSS) software (SPSS Inc., Armonk, NY, USA) was used for statistical analysis. Data are reported descriptively as frequency (%). A p-value <0.05 was used for statistical significance.

## Results

A total of 287 students responded to the survey. Of the 287 students, 50 (17.4%) students had prior patient exposure to TM, while 237 students (82.6%) did not. Meanwhile, 17.1% (N=49) of the students planned to utilize TM in the future, while 18.5% (N=53) students had no plans to utilize it, and 64.4% (N=185) remained undecided. Of those who had prior clinical experience with telemedicine, 32.0% (n=16) intended to utilize telemedicine in the future, while only 13.9% (n=33) of those who did not have clinical exposure planned to utilize it (Table 1). Of the undecided students, only 13.5% (N=25) had been previously exposed to telemedicine, while 86.5% (N=160) were never exposed.

**Table 1:** Prior Clinical Exposure to Telemedicine

Future plans to utilize telemedicine	Yes	No	Total
Plan to utilize	16	33	49 (17.1%)
Undecided	25	160	185 (64.4%)
No plans to utilize	9	44	53 (19.5%)
Total	50 (17.4%)	237 (82.6%)	

Regarding students’ opinions on the necessity to require telemedicine in their schools’ curricula, 35.2% (N=101) students thought that telemedicine should be a requirement, while 26.1% (N=75) thought it should not and 38.7% (N=111) remained undecided. Of note, only 19.5% of (N=56) students stated that didactics on telemedicine were offered by their medical school, while 37.6% (N=108) stated telemedicine was not offered and the majority of students (42.9%, N=123) were not sure whether it was offered. Of those who previously had didactics on telemedicine (N=56), 48.2% (n=27) thought telemedicine should be a requirement, while 26.8% (N=15) believed it should not be and 25.0% (n=14) remained undecided (Table 2). On the contrary, when telemedicine was not offered (N=108), only 25.9% (N=28) thought telemedicine should be part of the medical schools’ curriculum, while 31.5% (N=34) thought it should not be part of the curriculum and the majority of students (42.6%, N=46) remained undecided.

**Table 2:** Didactics on Telemedicine Offered

Telemedicine should be incorporated in medical school curriculum	Yes	No	Unsure	Total
Should be a requirement	27	28	46	101 (35.2%)
Undecided	14	46	51	111 (38.7%)
Should not be a requirement	15	34	26	75 (26.1%)
Total	56 (19.5%)	108 (37.6%)	123 (42.9%)	

In regard to intended residency, the most popular intended residencies included internal medicine (28.8%), pediatrics (22.9%), and emergency medicine (21.0%). Fifteen out of 17 choices for intended residency were correlated with a positive interest in utilizing telemedicine in their future practices (Table 3). Students who had indicated an interest in going into primary care demonstrated interests of 58.5% (family medicine), 50.0% (internal medicine), and 42.5% (pediatrics). The three intended residencies with the greatest interest levels were psychiatry (75.0%), pathology (66.7%), and dermatology (63.2%). On the contrary, the top three specialties with the highest disinterest percentage in the future use of telemedicine included urology (50.0%), ophthalmology (33.3%), and pediatrics (23.3%). The top three specialties with the highest percentage of whom responded as undecided were emergency medicine (41.8%), internal medicine (35.9%), and obstetrics and gynecology (34.6%).

**Table 3:** Interest Levels of Utilizing Telemedicine per Different Intended Residency Choices

Intended residency choice	Percentage of total medical students (n=287)	Interested	Disinterested	Undecided
Anesthesiology	11.0%	45.7%	20.0%	34.3%
Dermatology	6.0%	63.2%	21.1%	15.8%
Emergency Medicine	21.0%	43.3%	14.9%	41.8%
Family Medicine	16.6%	58.5%	13.2%	28.3%
Internal Medicine	28.8%	50.0%	14.1%	35.9%
Neurology	6.0%	42.1%	10.5%	47.4%
Obstetrics and Gynecology	16.3%	51.9%	13.5%	34.6%
Ophthalmology	4.7%	60.0%	33.3%	6.7%
Orthopedic Surgery	6.6%	42.9%	19.0%	38.1%
Otolaryngology	5.6%	44.4%	22.2%	33.3%
Pathology	4.7%	66.7%	13.3%	20.0%
Pediatrics	22.9%	42.5%	23.3%	34.2%
Pediatric sub-specialty	15.4%	46.9%	20.4%	32.7%
Psychiatry	7.5%	75.0%	12.5%	12.5%
Surgical sub-specialty	16.6%	47.2%	17.0%	35.8%
Urology	2.5%	12.5%	50.0%	37.5%
Other	10.7%	55.9%	17.6%	26.5%

Lastly, some of the students’ testimonials on their opinions on telemedicine were collected and have been included in this article (Table 4).

**Table 4:** Telemedicine Testimonials TM, telemedicine

	Students' opinions
In favor of TM use	“I am currently doing a dermatology rotation where this has been my first exposure. It is definitely a very valuable tool as some things can be easily diagnosed and prevents the patient from having to travel hours away (we cover 15 states). Other times we can’t diagnose by telemedicine and require face-to-face meetings. From my experience though it is a great resource”.
Undecided on TM use	“I don’t know much about telemedicine, but it seems like it would not allow me to have the same kind of relationship I would be able to have seeing patients in person”, “I have such limited exposure to it that I don’t yet have a clear opinion about it. I feel that this may be the way medicine is moving due to progress in technology, but I’m not sure if I’d personally prefer it over seeing a patient in person”.
Against TM use	“While a great asset for people that have no other means of communication with a provider, this form of medicine should not be used as a sole resource. It should always be used as an adjunct to periodic clinical visits. There is nothing that can replace the importance of the physical exam and more importantly the physician’s ability to ascertain the holistic presentation of a patient as in a physical encounter”.

## Discussion

Telemedicine is a rapidly growing field that supports long-distance healthcare services. It is defined by the World Health Organization as the use of electronic means for the prevention, diagnosis, and treatment of disease; research and evaluation; and education by healthcare providers to improve health [16]. It has been shown to improve access to healthcare at all levels, promote patient-centered care at lower costs, enhance efficiency in clinical decision making, increase the effectiveness of chronic disease management, and promote individual adoption of healthy lifestyles and self-care [17].

Patient outcomes and patient feasibility to engage in telehealth, as well as physician engagement involving in-patient and/or out-patient usage of telehealth modalities, have recently been critical areas of pilot research [18]. The importance and reliance on TM have only been strengthened with the rise of the coronavirus disease 2019 (COVID-19) pandemic, with many hospitals and clinics turning to telemedicine. TM has been used in various ways throughout the world to ease the burden on the healthcare system, including using telemedicine for non-urgent visits as well as allowing healthcare workers to check-in on patients remotely while minimizing contact even within hospitals [19-21]. Therefore, using a bottom-up approach by addressing the needs of physicians-in-training is necessary to understand the role that telehealth will play in the new era of globalized medicine [10].

With this rise in popularity and efficacy demonstrated by TM, the question arises whether medical schools should modify their curriculum to emphasize early exposure to TM through didactics and clinical experiences.

Our results suggest that while a significant number of US medical students are interested in TM, the majority of the students appear to be undecided. This may be due to a deficit in the US medical schools’ curricula in TM leading to students' inability to articulate well-informed opinions about TM. As seen from the results, roughly two-thirds of all surveyed students were undecided regarding their plans to utilize TM in the future. However, of those undecided students, 86.5% were never exposed to TM, which supports the presumption that it may be difficult to form an opinion on telemedicine when one was never educated on it. Furthermore, when students were exposed to TM in a clinical setting, the percentage of students interested in utilizing telemedicine in the future nearly doubled from 17.1% to 32%. As seen in the Results section, about two-thirds of all surveyed students were undecided regarding their plans to utilize TM. Of those, 86.5% were never exposed to TM, which supports the presumption that it may be difficult to form an opinion on a topic one was never educated on. These results suggest that the more clinical experience medical students have with telemedicine, the more favorable their opinions on telemedicine become, and the greater the likelihood of utilizing it in the future. This may be due to an increase in exposure as well as an increase in comfort levels with this advancing technology.

Similar trends were observed when asked whether medical schools’ curricula should be changed to require more training and introduction to telemedicine. Results from a previous study suggested that the implementation of a hands-on medical elective in telehealth can help medical students formulate opinions on their comfort and ability to utilize it [22]. Significantly, the number of students who wanted TM to be incorporated into the medical schools’ curricula was nearly 10% greater than those opposed to it. Furthermore, the percentage of those in favor of TM curricula grew to 48.2% from 35.2% when they were previously exposed to didactics on telemedicine. This suggests that the more clinical experience and education medical students have with TM, the more favorable their opinions on TM becomes. Furthermore, the lack of exposure, and ultimately awareness, may breed mixed opinions from students on whether TM should or should not be a requirement in a medical school.

Perhaps most interestingly, the results seem to suggest that utilization and interest in TM are not limited to primary care. In fact, the highest interest was demonstrated in specialties such as psychiatry, pathology, and dermatology. These three specialties may have exhibited the highest interest in TM as they require less physical examination that is hands-on and more of a visual and conversational nature. This may indicate the physical limitation of telemedicine, the inability to physically touch the patient and rather rely on the patient to “show” the doctor what is wrong. Nevertheless, the overwhelming interest in telemedicine among all intended specialties highlights the growing importance of TM and the need to provide increased education and clinical exposure to all students.

Admittedly, although the results of the study seem to suggest that more education and clinical exposure to TM should be incorporated into the medical schools’ curriculum, the cost-effectiveness and feasibility are not something that this study was able to address. Cost and feasibility are the issues that current healthcare providers who are using TM are facing; other challenges are the lack of organization, technical skills, lack of internet infrastructure or TM equipment, and inadequate financial support [9,23]. Additionally, TM users are concerned about ethical issues, patients’ data, and privacy [8]. More analysis in regard to the feasibility and cost-effectiveness of increasing TM training among medical students needs to be addressed in future studies.

As medicine continues to incorporate technology into the care of patients, it is important for training institutions to expose future physicians to the modalities of care they will be utilizing. Medical students have been shown to graduate not feeling prepared to utilize TM effectively [10]. It is clear that this is a rapidly growing area in medicine and more effort should be made to have greater training in the use of TM starting from medical school. To accomplish this, TM training needs to move forward to the level in which almost all the medical students have at least a basic understanding of the complex nature of TM and its socioeconomic and cultural principles [10,24]. While medical students’ exposure to TM will naturally occur as more and more physicians use it in their practice, it is an area that is expanding rapidly and can be intentionally incorporated in undergraduate medical education.

To our knowledge, and based on collected data, a multitude of medical schools including the University of Central Florida College of Medicine are implementing a telehealth elective or a clerkship. Medical students who participate in these electives consider it a useful educational tool and believe TM can potentially contribute to their medical knowledge, patient care skills, and system-based practice [25,26]. For example, the Cleveland Clinic has incorporated TM for second-year medical students to interview dialysis patients [27,28]. At Thomas Jefferson University, third- and fourth-year medical students can participate in a TM elective where they help patients and the medical team during rounds through TM [7].

Lastly, the industry projections for the global compounded annual growth rate of TM are 13%-27% with a market cap of greater than 180 billion US dollars by year 2026 [28,29].

This study was conducted prior to the COVID-19 pandemic. Thus, it may not reflect the most up-to-date sentiments about the usefulness of TM. With the current pandemic, more and more healthcare providers have turned towards telehealth as the main form of healthcare services. With this increased usage, it will be interesting to note any changes to perception towards TM in terms of its usefulness and its limitations. Additionally, although surveys were sent to 20 allopathic medical schools, the number of students who returned the surveys was limited to 287 students and the overall response rate was not significant. We believe the lack of direct access to all the medical students was one of the main limitations of this research work that can potentially lead to misinterpretation of the results. Also, this study did not assess the cost-effectiveness of TM in either medical curriculum or clinical practice.

## Conclusions

In this study, three key points of interest for physicians-in-training and their future curricula were elucidated through a survey aimed at determining attitudes and levels of exposure towards telemedicine. These key points were as follows: what are the opinions on curricula on TM, how does exposure to TM impact students’ interest in using telemedicine in the future, and what effect does a student’s intended residency have on his or her attitude towards TM. The results of this study suggest that most students do not receive adequate exposure to TM, which leads them to be unable to form more mature opinions on telemedicine. Increased early clinical exposure and education on telemedicine will benefit students’ preparedness and attitude towards telemedicine across all intended specialties.
